# Fuzzy analytic hierarchy process based generation management for interconnected power system

**DOI:** 10.1038/s41598-024-61524-2

**Published:** 2024-05-20

**Authors:** T. Varshney, A. V. Waghmare, V. P. Singh, V. P. Meena, R Anand, Baseem Khan

**Affiliations:** 1 Department of EECE, SSET, Sharda University, Greater Noida, Uttar Pradesh 201310 India; 2 Department of Electrical Engineering, Malaviya National Institute of Technology, Jaipur, Rajasthan 302017 India; 3 Department of Electrical and Electronics Engineering, Amrita School of Engineering, Bengaluru, Amrita Vishwa Vidyapeetham, Bengaluru, India; 4 Department of Electrical and Computer Engineering, Hawassa University, Hawassa, Ethiopia

**Keywords:** Fuzzy AHP, AHP, Jaya optimization algorithm, PID controller, AGC, Power system, Engineering, Electrical and electronic engineering, Energy infrastructure

## Abstract

Decision makers consistently face the challenge of simultaneously assessing numerous attributes, determining their respective importance, and selecting an appropriate method for calculating their weights. This article addresses the problem of automatic generation control (AGC) in a two area power system (2-APS) by proposing fuzzy analytic hierarchy process (FAHP), an multi-attribute decision-making (MADM) technique, to determine weights for sub-objective functions. The integral-time-absolute-errors (ITAE) of tie-line power fluctuation, frequency deviations and area control errors, are defined as the sub-objectives. Each of these is given a weight by the FAHP method, which then combines them into an single final objective function. This objective function is then used to design a PID controller. To improve the optimization of the objective function, the Jaya optimization algorithm (JOA) is used in conjunction with other optimization techniques such as sine cosine algorithm (SCA), Luus–Jaakola algorithm (LJA), Nelder–Mead simplex algorithm (NMSA), symbiotic organism search algorithm (SOSA) and elephant herding optimization algorithm (EHOA). Six distinct experimental cases are conducted to evaluate the controller’s performance under various load conditions, with data plotted to show responses corresponding to fluctuations in frequency and tie-line exchange. Furthermore, statistical analysis is performed to gain a better understanding of the effectiveness of the JOA-based PID controller. For non-parametric evaluation, Friedman rank test is also used to validate the performance of the proposed JOA-based controller.

## Introduction

Decision making is inherently complex and frequently requires the simultaneous consideration of multiple factors as well as expert judgment^1^. Because of this, the strategic identification of key factors becomes imperative. Analytic hierarchy process (AHP) has become a popular method for handling multi-attribute decision-making (MADM) scenarios. The reasons for the widespread use of AHP are:

- Its ease of use.
- Flexibility in incorporating a wide range of variables with quantitative and qualitative characteristics.
- Wide application in different fields.
- Accessibility of auxiliary software.

Parekh et al. demonstrated use of AHP to determine the relative importance of each performance indicator for solid waste management (SWM) in^2^. A land susceptibility model is developed in^3^ using AHP where relative weights of all landslip instability factors are determined with the help of AHP. AHP technique, along with GIS-based ranking were used in^4^ for finding the best locations in Kayseri, Turkey for solar photovoltaic (PV) power plant construction. Similar to this, Hammami et al. in^5^ also applied AHP technique along with GIS based multi-criteria decision analysis in flood susceptibility mapping at Tunisia. In^6^, based on AHP, the expert evaluation matrix is optimised using the accelerating genetic algorithm to determine the subjective weights. Nyimbili integrated AHP with TOPSIS and GIS techniques in^7^ for analyzing earthquake hazards. In^8^, weights of groundwater indicators, which are used for groundwater quality evaluation, were determined using AHP.

In spite of all the advantages of the AHP technique, the realization that traditional AHP has trouble in adequately addressing uncertainty^9^, especially when decision-makers are limited to a predetermined rating system from 1 to 9. This led to the adoption of fuzzy AHP (FAHP). FAHP is a derivative of AHP that has gained popularity recently and combines AHP with fuzzy set theory. Decision-makers have more freedom to have more flexible scales that use fuzzy membership functions and linguistic variables rather than deterministic or precise values in order to effectively capture uncertainty.

Many researchers have opted FAHP methods for decision making. Ertuğrul et al. in^10^ used FAHP technique for weight determination in facility location selection process. In^11^, FAHP is used in determining weights of machine tool alternatives for multiple attributes. Shaygan, in^12^, illustrated an actual implementation of FAHP method for identifying, ranking, and choosing enhancement projects for a hospital’s under performing appointment system. In^13^, FAHP method is implemented to fully structured decision-making problems involving alternatives, sub-criteria, and criteria for evaluating water management plans in a section of Brazil’s Paraguacu river basin. In^14^, three potential landfill locations for Istanbul are assessed using FAHP in addition to expert opinion. Similarly, Karasan et al. in^15^ implemented novel pythagorean FAHP method landfill site selection problem.

MADM techniques like rank sum weight, AHP and rank exponent are implemented for AGC problem of 2-APS in the literature^16–19^. AGC ensures maintaining the overall power balance of the system that is dependent on critical factors such as frequency deviation, area control errors, and tie-line power deviation^20,21^. The controllers used in AGC play an important role in correcting system imbalances^22–24^. It is critical to optimize the parameters of these controllers to ensure a consistent and efficient flow of power. When designing controllers, choosing an appropriate objective function is necessary for improving and optimizing^25,26^ parameter changes. This objective function typically includes sub-objective functions that represent error indices for frequency deviation, area control errors, and tie-line power deviation. Prioritizing sub-objective functions and assigning appropriate ranks and weights are critical in attaining an optimal outcome for the objective function. As a result, once the weights are properly determined, additional step involving optimization^27–30^ of the objective function is required.

In this paper, FAHP technique is implemented for AGC problem of 2-APS. This technique is used to provide weights corresponding to sub-objective functions, which are further utilized to tune PID controller. For sub-objective functions, the ITAE evaluations of the frequency deviations, control errors, and lie-line power deviation for the AGC problem of 2-APS are taken into account. The PID controller design utilizes each of these sub-objective functions. Subsequently all weighted sub-objectives are combined to create the objective function and further Jaya optimization algorithm (JOA)^31–33^ is used to minimize the same. Six different experimental cases are used to evaluate the FAHP’s effectiveness. Additionally, sine cosine algorithm (SCA), Luus–Jaakola algorithm (LJA), Nelder-Mead simplex algorithm (NMSA), symbiotic organism search algorithm (SOSA) and elephant herding optimization algorithm (EHOA) are used in optimization to demonstrate the effectiveness of the JOA algorithm-based controller. The results are compared for each of the six load variations, and both tabular and graphical comparisons are displayed. Comparisons are made on the basis of specifications such as peak overshoots, settling times, decision parameters, and values of the objective function. A statistical analysis is performed by evaluating mean, minimum, maximum, and standard deviation values of the objective function obtained from JOA, SCA, LJA, NMSA, SOSA, and EHOA. Friedman rank test is used to further elucidate the effectiveness and accuracy of the results obtained. By assigning a mean rank to each of the six algorithms and calculating an overall *Q* value and *p* value, this test offers a non-parametric analysis.

The key objectives of this article are as follows:

- FAHP technique is investigated to determine weights of sub-objective functions for AGC problem of 2-APS.
- ITAEs of frequency deviations, tie-line deviations and control errors are considered as sub-objective functions.
- PID controller is designed on the basis of single objective function formed by merging all the weighted sub-objective functions.
- Minimization of objective function is performed using JOA.
- The results obtained from JOA are compared with those obtained from SCA, LJA, NMSA, SOSA and EHOA, by performing statistical analysis and Friedman rank test.

The architecture of this study is structured as follows. Section “Fuzzy analytic hierarchy process” ellaborates the FAHP method in detail. The considerated 2-APS is introduced in Sect. “Considerated system”. The implementation of FAHP method to 2-APS is provided in Sect. “Implementation of FAHP method for AGC problem”. In Sect. “Jaya optimization algorithm”, Jaya optimization algorithm is explained. In Sect. “Results and discussions”, the results obtained are discussed in detail. Finally the derived conclusion is presented in Sect. “Conclusion”.

## Fuzzy analytic hierarchy process

Fuzzy analytic hierarchy process (FAHP) is a multi attribute decision making (MADM) technique used to determine weights using fuzzy rules^34^. On the basis of these fuzzy rules, a decision matrix is formed whose elements denote the performance measure of one decision making problem with respect to other decision making problem. These performance measures are dependant on fuzzy membership function. For defining fuzzy rules, triangular fuzzy membership (TFM)^35^ function with real numbers are utilized. Let $F_{\phi }$ be TFM function consisting of triangular fuzzy rules, which is defined in (1).

1 $$\begin{aligned} F_{\phi }=\left\{ \begin{aligned} 0&\qquad \phi <L \\ (\phi -L)/(M-L)&\qquad L \le \phi \le M \\ (U-\phi )/(U-M)&\qquad M \le \phi \le U\\ 0&\qquad \phi >U \end{aligned}\right. \end{aligned}$$

In (1), *L*, *M* and *U* denotes the lower, middle and upper range values of TFM function element. For each fuzzy element of a TFM function, a fuzzy performance index is defined according to its significance level, as given in Table 1.

**Table 1:** Performance indices with TFM function and their relative significance.

Fuzzy	Fuzzy element as a	Significance
Performance index	TFM function	Level
10	(9,10,11)	Absolute
9	(8,9,10)	Highest
8	(7,8,9)	Higher
7	(6,7,8)	High
6	(5,6,7)	Above average
5	(4,5,6)	Average
4	(3,4,5)	Low
3	(2,3,4)	Lower
2	(1,2,3)	Least
1	(1,1,1)	Identical

**Figure 1:**
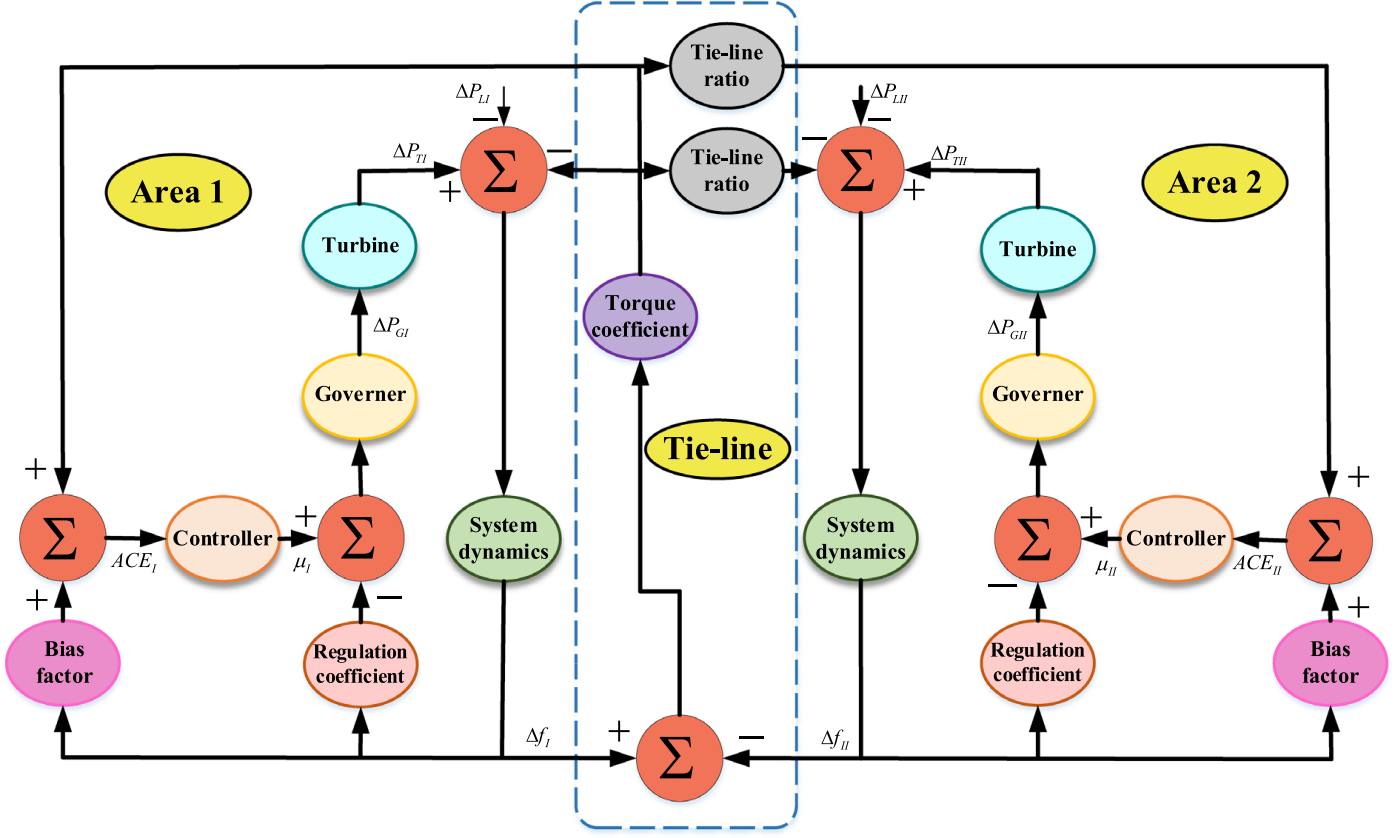
2-APS.

**Table 2:** 2-APS parameters (Area 1).

2-APS parameters (Area 1)		
Parameter	Variable	Value (unit)
Frequency	*f*	60Hz
Frequency deviation	$\Delta {f_{I}}$	–
Area control error	$ACE_{I}$	–
Bias factor	$\beta _{I}$	0.05pu MW/Hz
Control input	$\mu _{I}$	–
Governer’s speed regulation constant	$R_{I}$	2.4Hz/pu
Governer’s time constant	$\tau _{GI}$	0.08sec.
Turbine’s time constant	$\tau _{TI}$	0.3sec
System’s gain constant	$K_{I}$	120Hz/pu MW
System’s time constant	$\tau _{I}$	20sec.
Governer power deviation	$\Delta {P_{GI}}$	–
Turbine power deviation	$\Delta {P_{TI}}$	–
System’s load change	$\Delta {P_{LI}}$	–

**Table 3:** 2-APS parameters (Area 2).

2-APS parameters (Area 2)		
Parameter	Variable	Value (unit)
Frequency	*f*	60Hz
Frequency deviation	$\Delta {f_{II}}$	–
Area control error	$ACE_{II}$	–
Bias factor	$\beta _{II}$	0.05pu MW/Hz
Control input	$\mu _{II}$	–
Governer’s speed regulation constant	$R_{II}$	2.4Hz/pu
Governer’s time constant	$\tau _{GII}$	0.08sec.
Turbine’s time constant	$\tau _{TII}$	0.3sec
System’s gain constant	$K_{II}$	120Hz/pu MW
System’s time constant	$\tau _{II}$	20sec.
Governer power deviation	$\Delta {P_{GII}}$	–
Turbine power deviation	$\Delta {P_{TII}}$	–
System’s load change	$\Delta {P_{LII}}$	–

The detailed procedure involved in determining weights using FAHP is discussed below:

**Step 1: Identification of criteria and their relative siginifances**

In FAHP, criteria are needed to be defined for decision making, which are termed are alternatives and attributes. Let there be *N* alternatives and *M* attributes. The weights corresponding to attributes are denoted as $O_m$, where $m=1,2,\cdots ,M$, and those corresponding to alternatives are denoted as $O_n$, where $n=1,2,\cdots ,N$.

**Step 2: Pair-wise decision matrix formulation**

After defining the alternatives and attributes, a pair wise decision matrix is formed using TFM function. The elements of matrix are fuzzy element taken from Table 1 and are denoted by $P_{mn}$ and their significance level is decided on the basis of $m^{th}$ attribute’s relation with $n^{th}$ alternative. For example, if $m^{th}$ attribute is at “Highest” significance level with respect to $n^{th}$ alternative, then $P_{m,n}$ will be “(8,9,10)” which is considered from Table 1. And if there is no difference in the significance level of $m^{th}$ attribute and $n^{th}$ alternative, then then $P_{mn}$ will be considered as ’Identical’ i.e. “(1,1,1)”. The representation of decision matrix is given in (2). FAHP based decision matrix

2 $$\begin{aligned} A= \begin{matrix} O_1 \\ O_2 \\ O_3 \\ \vdots \\ O_{M} \end{matrix} \quad {\mathop { \begin{bmatrix} P_{1,1} &{} P_{1,2} &{} P_{1,3} &{}\cdots &{} P_{1,N}\\ P_{2,1} &{} P_{2,2} &{} P_{2,3} &{} \cdots &{} P_{2,N}\\ P_{3,1} &{} P_{3,2} &{} P_{3,3} &{} \cdots &{} P_{3,N}\\ \vdots &{} \vdots &{} \vdots &{} \ddots &{} \vdots \\ P_{M,1} &{} P_{M,2} &{} P_{M,3} &{} \cdots &{} P_{M,N} \end{bmatrix}}\limits ^{ O_1 \qquad O_2 \qquad O_3 \qquad \cdots \quad O_{N}}} \end{aligned}$$

Fuzzy element $P_{m,n}$ i.e. TFM function is defined such that $P_{m,n} = P^{-1}_{m,n}$ when $m \ne n$ and $P_{m,n} = 1$ when $m = n$.

**Step 3: Evaluation of geometric mean**

The interval arithmetic for TFM function is utilized to evaluate geometric mean ($GM_{m}$) of the $m^{th}$ alternative which is calculated using (3).

3 $$\begin{aligned} GM_{m}=\Bigg [\prod _{n=1}^{N}P_{m,n}\Bigg ]^{{1\backslash N}} \end{aligned}$$

where, $GM_{m}$ is geometric mean and it shows radical root of $m^{th}$ alternative’s in decision matrix.

**Step 4: Calculation of fuzzy weights**

For respective attributes, relative fuzzy weights $(FO_m)$ are calculated as

4 $$\begin{aligned} FO_{m}=\frac{GM_{m}}{\sum _{m=1}^{M}GM_{m}} \end{aligned}$$

**Step 5: Calculation of best non-fuzzy performance value as weights**

The calculation of best non-fuzzy performance (BNFP) value as weights is done as

5 $$\begin{aligned} O_{m}=\frac{[FO(L)_{m}+FO(M)_{m}+FO(U)_{m}]}{3} \end{aligned}$$

where $FO(L)_{m}$, $FO(M)_{m}$ and $FO(U)_{m}$ represent the lower, middle and upper fuzzy values, respectively, to calculate BNFP value based on fuzzy membership function.

## Considerated system

A schematic representation of two area power system (2-APS), inspired from^36^, is provided in Fig. 1. With two thermal power plants contributing 1000 MW each to the overall load, the system configuration includes a combined capacity of 2000 MW. This configuration simulates an actual networked power system. The parameters of 2-APS for area 1, area 2 and tie-line region are described in Table 2, Table 3 and Table 4. The mathematical models of components of 2-APS for area 1 and area 2, in form of transfer functions, are presented in Table 5.

**Table 4:** 2-APS parameters (Tie line).

2-APS parameters (tie line)		
Parameter	Variable	Value (unit)
Torque coefficient	$T_{tie-line}$	0.545 pu
Tie-line ratio	$A_{tie-line}$	-1
Tie-line power deviation	$\Delta {Z_{tie-line}}$	-

**Table 5:** 2-APS transfer functions.

2-APS transfer functions		
Component	Area 1	Area 2
Turbine	$TF_{TI}=\frac{1}{1+s\tau _{TI}}$	$TF_{TII}=\frac{1}{1+s\tau _{TII}}$
Generator	$TF_{GI}=\frac{1}{1+s\tau _{GI}}$	$TF_{GII}=\frac{1}{1+s\tau _{GII}}$
System dynamics	$TF_{I}=\frac{K_{I}}{1+s\tau _{I}}$	$TF_{II}=\frac{K_{II}}{1+s\tau _{II}}$

**Table 6:** 2-APS parameters (constraints).

2-APS parameters (constraints)		
Parameter	Max value	Min value
Proportional gain	$\Gamma _P^{max}=3$	$\Gamma _P^{min}=0$
Integral gain	$\Gamma _I^{max}=3$	$\Gamma _I^{min}=0$
Derivative gain	$\Gamma _D^{max}=3$	$\Gamma _D^{min}=0$
Filter	$F^{max}=500$	$F^{min}=0$

### PID controller

PID controllers are extensively used by numerous industries, especially in process industries. Their extensive use is because of their dependability and ease for handling. For the majority of systems, they offer strong and dependable performance as long as the PID parameters are chosen or adjusted to guarantee an acceptable closed-loop performance. PID controller operates on the basis that each of the three terms i.e. proportional, integral, and derivative are supposed to be “tuned” appropriately. An input is subjected to a correction factor i.e. error, which is computed based on the variation between these values. A basic PID controller is mathematically expressed in (6).

6 $$\begin{aligned} Y(s)= \Gamma _{P}*E(s)+\frac{\Gamma _{I}}{s}*E(s)+\Gamma _{D}s*E(s) \end{aligned}$$

In (6), *Y*(*s*) is output signal; $\Gamma _{P}$, $\Gamma _{I}$ and $\Gamma _{D}$ are proportional, integral, and derivative terms, respectively; and *E*(*s*) is error signal. To lessen the effect of noise, the PID controller used in this study integrates a filter *F* with the derivative gain. The generated outputs are control inputs, $\mu _{I}$ and $\mu _{II}$, and area control errors $ACE_{I}$ and $ACE_{II}$ are controller inputs. The expressions for $\mu _{I}$ and $\mu _{II}$ are presented in (7) and (8).

7 $$\begin{aligned} \mu _{I}(s)= & {} \Gamma _{P}*ACE_{I}(s)+\frac{\Gamma _{I}}{s}*ACE_{I}(s)+\Gamma _{D}s\frac{1}{\frac{1}{s}+\frac{1}{F}}*ACE_{I}(s) \end{aligned}$$

8 $$\begin{aligned} \mu _{II}(s)= & {} \Gamma _{P}*ACE_{II}(s)+\frac{\Gamma _{II}}{s}*ACE_{II}(s)+\Gamma _{D}s\frac{1}{\frac{1}{s}+\frac{1}{F}}*ACE_{II}(s) \end{aligned}$$

$ACE_{I}$ and $ACE_{II}$ from (7) and (8) can be further elaborated as follows:

9 $$\begin{aligned} ACE_{I}(s)= & {} \Delta {Z_{tie-line}}(s)+\beta _{I}.\Delta {f_{I}}(s) \end{aligned}$$

10 $$\begin{aligned} ACE_{II}(s)= & {} -A_{tie-line}.\Delta {Z_{tie-line}}(s)+\beta _{II}.\Delta {f_{II}}(s) \end{aligned}$$

### Objective function formulation

Maintaining area control errors to minimum values, maintaining tie-line powers, and upholding the frequencies of area 1 and area 2 are essential in ensuring a balanced power flow. Sub-objective functions are developed by taking these three factors into account, which are further clubbed into a single objective function. For this study, ITAEs of aforementioned factors are, respectively, evaluated to determine the sub-objective functions, which are mathematically presented in (11), (12) and (13).

11 $$\begin{aligned} OF_{I}= & {} \int _0^{T_{st}}\Delta {f_{I}}tdt+\int _0^{T_{st}}\Delta {f_{II}}tdt \end{aligned}$$

12 $$\begin{aligned} OF_{II}= & {} \int _0^{T_{st}}ACE_{I}tdt+\int _0^{T_{st}}ACE_{II}tdt \end{aligned}$$

13 $$\begin{aligned} OF_{III}= & {} \int _0^{T_{st}}\Delta {Z_{tie-line}}tdt \end{aligned}$$

where,

$OF_{I}$: ITAEs of frequency deviations;

$OF_{II}$: ITAEs of area control errors;

$OF_{III}$: ITAE of tie-line power deviation;

$T_{st}$: Total simulation time.

These sub-objectives are provided weights depending on their importance and a single objective function is developed combining all three weighted sub-objectives. The structure of developed objective function is given as

14 $$\begin{aligned} OF=(O_1)(OF_{I})+(O_2)(OF_{II})+(O_3)(OF_{III}) \end{aligned}$$

where, $O_1$, $O_2$ and $O_3$ are weights of $OF_{I}$, $OF_{II}$ and $OF_{III}$, respectively.

## Implementation of FAHP method for AGC problem

In this contribution, for determining the weights associated with sub-objective functions, FAHP method is implemented, following the steps priorly discussed in Sect. “Fuzzy analytic hierarchy process”. The sub-objectives (11), (12) and (13) are signified with highest, higher and above average significance level, respectively. Corresponding to these significance levels, the weights are calculated using FAHP method discussed in Sect. “Fuzzy analytic hierarchy process”. Here, the sub-objectives $OF_I$, $OF_{II}$ and $OF_{III}$ are considered as attributes as well as alternatives. Therefore, the value of *M* and *N* will be same, i.e 3, for this problem. The significance level of one sub-objective with respect to other, is assigned as follows: (Table 6)

- $OF_I$ is at “Highest” significance level with respect to $OF_{III}$.
- $OF_I$ is at “Higher” significance level with respect to $OF_{II}$.
- $OF_{II}$ is at “Above average” significance level with respect to $OF_{III}$.

Considering these significance levels, Table 1 is further transformed to Table 7. Corresponding to the fuzzy elements presented in Table 7 and referring to (2), (15) is generated.

**Table 7:** Performance indices with TFM function and their relative significance.

Fuzzy	Fuzzy element as a	Significance
Performance index	TFM function	Level
9	(8,9,10)	Highest
8	(7,8,9)	Higher
6	(5,6,7)	Above average
1	(1,1,1)	Identical

15 $$\begin{aligned} A= \begin{matrix} OF_1 \\ OF_2 \\ OF_3 \\ \end{matrix} \quad {\mathop { \begin{bmatrix} (1,1,1) &{} (7,8,9) &{} (8,9,10)\\ (\frac{1}{7},\frac{1}{8},\frac{1}{9}) &{} (1,1,1) &{} (5,6,7)\\ (\frac{1}{8},\frac{1}{9},\frac{1}{10}) &{} (\frac{1}{5},\frac{1}{6},\frac{1}{7}) &{} (1,1,1)\\ \end{bmatrix}}\limits ^{ OF_1 \qquad OF_2 \qquad OF_3}} \end{aligned}$$

By exploiting (3), geometric means ($GM_{i}$) are calculated using as

16 $$\begin{aligned} \begin{aligned} GM_1&=[1\times 9\times 10]^{1/3}=4.4814\\ GM_2&=[1/9\times 1\times 7]^{1/3}=0.9196\\ GM_3&=[1/10\times 1/7\times 1]^{1}=0.2426\\ \end{aligned} \end{aligned}$$

The cumulative geometric mean is determined as

17 $$\begin{aligned} \sum _{i=1}^{N}GM_i=4.4814+0.9196+0.2426=5.6437 \end{aligned}$$

The fuzzy weights $FO_{U1}$, $FO_{U2}$ and $FO_{U3}$, are calculated with the help of (4) by utilizing the values from (16) and (17) as

18 $$\begin{aligned} \begin{aligned} FO_{U1}&=0.7633\\ FO_{U2}&=0.1783\\ FO_{U3}&=0.0583 \end{aligned} \end{aligned}$$

The values of fuzzy weights obtained in (18) are calculated by taking upper range values into consideration. Similarly lower and middle range values are calculated which are presented in (19) and (20), respectively.

19 $$\begin{aligned} FO_{L1}&=0.7941\nonumber \\ FO_{L2}&=0.1630\nonumber \\ FO_{L3}&=0.0430 \end{aligned}$$

20 $$\begin{aligned} FO_{M1}&=0.7800\nonumber \\ FO_{M2}&=0.1704\nonumber \\ FO_{M3}&=0.0496 \end{aligned}$$

The calculation of best non-fuzzy performance (BNFP) value as weight is done by utilizing (5) Weights for SOROEV model using FAHP

21 $$\begin{aligned} \begin{aligned} O_1&=0.7791\\ O_2&=0.1706\\ O_3&=0.0503 \end{aligned} \end{aligned}$$

Substituting these weights in overall objective function, (14) can be updated as follows:

22 $$\begin{aligned} OF=0.7791(OF_{I})+0.1706(OF_{II})+0.0503(OF_{III}) \end{aligned}$$

By putting values of $OF_{I}$, $OF_{II}$ and $OF_{III}$ from (11), (12) and (13), respectively, in (22), it is further modified to:

23 $$\begin{aligned} OF=0.7791\Bigg (\int _0^{T_{st}}\Delta {f_{I}}tdt+\int _0^{T_{st}}\Delta {f_{II}}tdt\Bigg )+0.1706\Bigg (\int _0^{T_{st}}ACE_{I}tdt+\int _0^{T_{st}}ACE_{II}tdt\Bigg )+0.0503\Bigg (\int _0^{T_{st}}\Delta {Z_{tie-line}}tdt\Bigg ) \end{aligned}$$

In order to optimize (23), it becomes necessary to define its boundary constraints. The subjected boundary constraints are defined as follows:

24 $$\begin{aligned} \begin{array}{cc} &{} \Gamma _P^{min}<\Gamma _P<\Gamma _P^{max}\\ &{} \Gamma _I^{min}<\Gamma _I<\Gamma _I^{max}\\ &{} \Gamma _D^{min}<\Gamma _D<\Gamma _D^{max}\\ &{} F^{min}<F<F^{max} \end{array} \end{aligned}$$

To optimize objective function (23), subjected to constraints shown in (24), Jaya algorithm is implemented, which is discussed further in Sect. “Jaya optimization algorithm”.

## Jaya optimization algorithm

The Jaya optimization algorithm(JOA) is inspired by the Sanskrit word of “victory”, represented as “Jaya”. It was first developed to solve both constrained and unconstrained optimization problems^37,38^. By eliminating less effective solutions, solutions within the Jaya population tend to converge towards the global optimum, mirroring the concept of “survival of the fittest” found in nature. Interestingly, this algorithm relies only on the total number of iterations and the size of the population. It does not require any particular controlling parameters.

The basic structure of working of JOA can be described in the below-mentioned five phases:

1. *Initialization* The process is started by initializing a set of population comprising of candidate solutions. Usually, these solutions are shown as vectors in the optimization problem’s search space.
2. *Determination* Utilizing the optimization problem’s objective function, determine candidate solution. Each solution is assigned with a fitness value by the objective function. This fitness value evaluates performance of the candidate corresponding to problem.
3. *Regeneration* For each iteration, the candidate solution is regenerated such that it moves towards the best solution (solution having lowest fitness value), ignoring the worst solution (solution having highest fitness value).
4. *Discontinuation* Once the predetermined number of iterations is completed or termination criterion is satisfied, the process is discontinued.
5. *Final solution* The solution obtained just before discontinuing the process is termed as the final solution.

**Figure 2:**
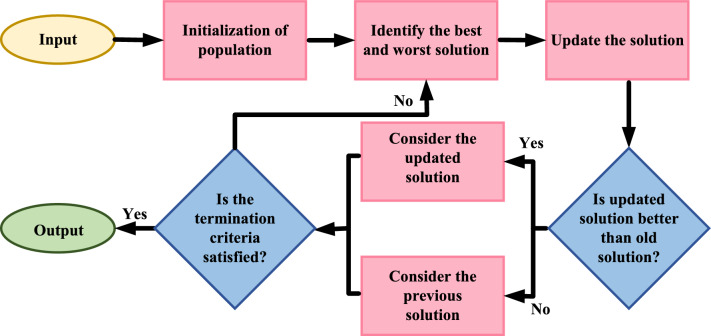
Jaya optimization.

The flowchart for JOA is provided in Fig. 2. In JOA, the expression for updated solution is represented as follows:

25 $$\begin{aligned} {\mathcal {O}}^{i^{'}}_{x,y}={\mathcal {O}}^{i}_{x,y}+{\mathcal {O}}_{1}+{\mathcal {O}}_{2} \end{aligned}$$

where,

26 $$\begin{aligned} \left. \begin{array}{ll} {\mathcal {O}}_{1}=\alpha _{1}( {\mathcal {O}}^{i}_{best,b}- {\mathcal {O}}^{i}_{x,y})\\ {\mathcal {O}}_{2}=-\alpha _{2}( {\mathcal {O}}^{i}_{worst,b}- {\mathcal {O}}^{i}_{x,y}) \\ \end{array} \right\} \end{aligned}$$

In (25), ${\mathcal {O}}^{i^{'}}_{x,y}$ and ${\mathcal {O}}^{i}_{x,y}$ are updated solution and current solution respectively, for $x^{th}$ candidate and $y^{th}$ decision parameter. While, ${\mathcal {O}}^{i}_{best,y}$ and ${\mathcal {O}}^{i}_{worst,y}$ in (26) denote best and worst solution,, respectively and $\alpha _{1}$ and $\alpha _{2}$ are random variables. An updated solution is produced after every iteration. If this revised solution performs better than the original, it will be considered for further iterations.

**Table 8:** Step load deviations.

Step load deviations		
Experimental cases	Area 1	Area 2
I	0.032	0
II	0	0.032
III	0.032	0.032
IV	0.032	-0.032
V	0.032	0.064
VI	0.064	0.032

**Table 9:** Results for case analysis I.

		JOA	SCA	LJA	NMSA	SOSA	EHOA
**Fitness**	*OF*	0.01770	0.03335	0.02262	0.04087	0.02609	0.02641
	$OF_{I}$	0.02044	0.03879	0.02605	0.04716	0.03027	0.03066
	$OF_{II}$	0.00536	0.00942	0.00762	0.01296	0.00799	0.00803
	$OF_{III}$	0.00879	0.01561	0.01139	0.02035	0.01231	0.01244
Decision parameters	$\Gamma _P$	1.88711	1.76927	2.87765	2.13089	2.15094	2.22966
	$\Gamma _I$	2.98252	2.49641	2.87080	2.00521	2.67468	2.72156
	$\Gamma _D$	0.52462	1.15719	1.14092	1.24554	1.12535	1.20696
	*F*	356.830	433.202	273.225	177.700	347.199	349.361
Settling time (s)	$\Delta {f_{I}}$	2.21656	4.16017	2.25456	2.99855	3.51192	3.63688
	$\Delta {f_{II}}$	3.23523	5.00380	4.36233	4.89006	3.46292	3.48560
	$\Delta {Z_{tie-line}}$	3.14423	5.41283	4.49587	4.99221	3.64359	3.65955
Peak overshoots (p.u.)	$\Delta {f_{I}}$	0.03119	0.02229	0.02180	0.02155	0.02238	0.02156
	$\Delta {f_{II}}$	0.01626	0.00998	0.00900	0.00906	0.00973	0.00913
	$\Delta {Z_{tie-line}}$	0.00541	0.00377	0.00314	0.00356	0.00351	0.00334

**Table 10:** Results for case analysis II.

		JOA	SCA	LJA	NMSA	SOSA	EHOA
Fitness	*OF*	0.01731	0.03335	0.02262	0.04086	0.02609	0.02641
	$OF_{I}$	0.01996	0.03879	0.02605	0.04715	0.03028	0.03066
	$OF_{II}$	0.00575	0.00942	0.00762	0.01296	0.00799	0.00803
	$OF_{III}$	0.00858	0.01561	0.01139	0.02035	0.01231	0.01244
Decision parameters	$\Gamma _P$	2.16060	1.76927	2.87765	2.13089	2.15094	2.22966
	$\Gamma _I$	2.99912	2.49641	2.87080	2.00521	2.67468	2.72156
	$\Gamma _D$	0.64784	1.15719	1.14092	1.24554	1.12535	1.20696
	*F*	470.811	433.202	273.225	177.700	347.199	349.361
Settling time (s)	$\Delta {f_{I}}$	3.29438	5.00379	4.36268	4.88837	3.46291	3.48559
	$\Delta {f_{II}}$	1.96761	4.15989	2.25635	2.99978	3.51762	3.64273
	$\Delta {Z_{tie-line}}$	3.44820	5.41284	4.49579	4.99238	3.64359	3.65957
Peak overshoots (p.u.)	$\Delta {f_{I}}$	0.01402	0.00998	0.00900	0.00906	0.00973	0.00913
	$\Delta {f_{II}}$	0.02841	0.02229	0.02180	0.02155	0.02238	0.02156
	$\Delta {Z_{tie-line}}$	0.00471	0.00377	0.00314	0.00356	0.00351	0.00334

**Table 11:** Results for case analysis III.

		JOA	SCA	LJA	NMSA	SOSA	EHOA
Fitness	*OF*	0.02541	0.06769	0.03822	0.05151	0.05128	0.04371
	$OF_{I}$	0.02984	0.07949	0.04488	0.06048	0.06022	0.05132
	$OF_{II}$	0.00000	0.00000	0.00000	0.00000	0.00000	0.00000
	$OF_{III}$	0.01268	0.03378	0.01908	0.02571	0.02559	0.02181
Decision parameters	$\Gamma _P$	2.16060	1.76927	2.87765	2.13089	2.15094	2.22966
	$\Gamma _I$	2.95342	2.77400	2.61036	2.29569	2.54692	2.89826
	$\Gamma _D$	0.52539	0.93834	0.78534	0.87023	1.24136	1.23978
	*F*	260.322	275.477	351.305	228.395	365.123	490.703
Settling time (s)	$\Delta {f_{I}}$	2.17045	4.03241	2.35049	2.67387	3.26389	3.85170
	$\Delta {f_{II}}$	2.17045	4.03241	2.35049	2.67387	3.26389	3.85170
	$\Delta {Z_{tie-line}}$	0.00000	0.00000	0.00000	0.00000	0.00000	0.00000
Peak overshoots (p.u.)	$\Delta {f_{I}}$	0.03532	0.02857	0.03006	0.02894	0.02296	0.02298
	$\Delta {f_{II}}$	0.03532	0.02857	0.03006	0.02894	0.02296	0.02298
	$\Delta {Z_{tie-line}}$	0.00000	0.00000	0.00000	0.00000	0.00000	0.00000

**Table 12:** Results for case analysis IV.

		JOA	SCA	LJA	NMSA	SOSA	EHOA
Fitness	*OF*	0.02403	0.03794	0.02752	0.03111	0.02888	0.02533
	$OF_{I}$	0.02609	0.03723	0.02890	0.03188	0.03016	0.02672
	$OF_{II}$	0.01302	0.02839	0.01627	0.02018	0.01783	0.01524
	$OF_{III}$	0.01788	0.04398	0.02452	0.03079	0.02628	0.02192
Decision parameters	$\Gamma _P$	2.46482	2.34051	2.54515	2.47623	2.32523	2.54709
	$\Gamma _I$	2.98858	1.94360	2.94214	2.52240	2.57513	2.835346
	$\Gamma _D$	1.00376	0.84323	1.57115	1.58464	1.28716	1.25174
	*F*	398.739	467.817	379.625	205.922	369.009	469.840
Settling time (s)	$\Delta {f_{I}}$	3.51062	4.40854	3.95176	4.33906	4.01833	3.82893
	$\Delta {f_{II}}$	3.51062	4.40854	3.95176	4.33906	4.01833	3.82893
	$\Delta {Z_{tie-line}}$	3.70408	6.00784	5.49316	4.22087	4.00612	3.93501
Peak overshoots (p.u.)	$\Delta {f_{I}}$	0.02183	0.02357	0.01773	0.01791	0.01960	0.01971
	$\Delta {f_{II}}$	0.02183	0.02357	0.01773	0.01791	0.01960	0.01971
	$\Delta {Z_{tie-line}}$	0.00715	0.00819	0.00594	0.00617	0.00652	0.00621

**Table 13:** Results for case analysis V.

		JOA	SCA	LJA	NMSA	SOSA	EHOA
Fitness	*OF*	0.03877	0.04128	0.06272	0.07654	0.05452	0.05560
	$OF_{I}$	0.04515	0.04797	0.07318	0.08939	0.06373	0.06498
	$OF_{II}$	0.00521	0.00532	0.00792	0.00878	0.00690	0.00697
	$OF_{III}$	0.01951	0.02134	0.03110	0.03780	0.02649	0.02712
Decision parameters	$\Gamma _P$	1.62401	1.59717	1.63668	2.67985	1.93025	2.06716
	$\Gamma _I$	2.92798	2.88402	2.32600	2.63987	2.68320	2.72282
	$\Gamma _D$	0.48128	0.43335	0.48760	1.32552	0.76239	0.82656
	*F*	355.622	218.227	369.819	440.721	300.932	228.823
Settling time (s)	$\Delta {f_{I}}$	2.38233	2.45129	3.36826	3.97366	2.87172	3.01707
	$\Delta {f_{II}}$	2.10890	2.16707	2.53177	3.18600	2.18507	2.23903
	$\Delta {Z_{tie-line}}$	3.05619	3.18510	3.68247	4.47757	3.46559	3.58744
Peak overshoots (p.u.)	$\Delta {f_{I}}$	0.04554	0.04734	0.04591	0.02496	0.03652	0.03449
	$\Delta {f_{II}}$	0.06934	0.07210	0.06923	0.04199	0.05680	0.05451
	$\Delta {Z_{tie-line}}$	0.00587	0.00616	0.00592	0.00302	0.00453	0.00424

**Table 14:** Results for case analysis VI.

		JOA	SCA	**LJA**	**NMSA**	**SOSA**	**EHOA**
Fitness	*OF*	0.04058	0.08797	0.08657	0.07043	0.08278	0.09451
	$OF_{I}$	0.04739	0.10230	0.10111	0.08229	0.09664	0.11049
	$OF_{II}$	0.00555	0.00996	0.00964	0.00846	0.00971	0.00957
	$OF_{III}$	0.01980	0.04555	0.04285	0.03453	0.04107	0.04659
Decision parameters	$\Gamma _P$	1.81290	2.02888	2.94046	1.97933	1.86144	2.79796
	$\Gamma _I$	2.90251	2.26469	2.60156	2.44598	2.17972	2.98638
	$\Gamma _D$	0.53182	0.33129	1.57187	0.91227	0.51777	2.14128
	*F*	199.643	256.339	269.611	433.240	393.858	410.815
Settling time (s)	$\Delta {f_{I}}$	1.64124	3.88906	3.59934	2.55364	3.02945	5.43786
	$\Delta {f_{II}}$	2.46416	4.49774	4.40004	3.28118	4.06844	6.06677
	$\Delta {Z_{tie-line}}$	3.13768	5.30282	4.86206	3.85088	4.41652	6.56501
Peak overshoots (p.u.)	$\Delta {f_{I}}$	0.06616	0.07505	0.03835	0.05207	0.06662	0.03223
	$\Delta {f_{II}}$	0.04276	0.04817	0.02229	0.03351	0.04349	0.02036
	$\Delta {Z_{tie-line}}$	0.00546	0.00632	0.00284	0.00409	0.00556	0.00266

**Figure 3:**
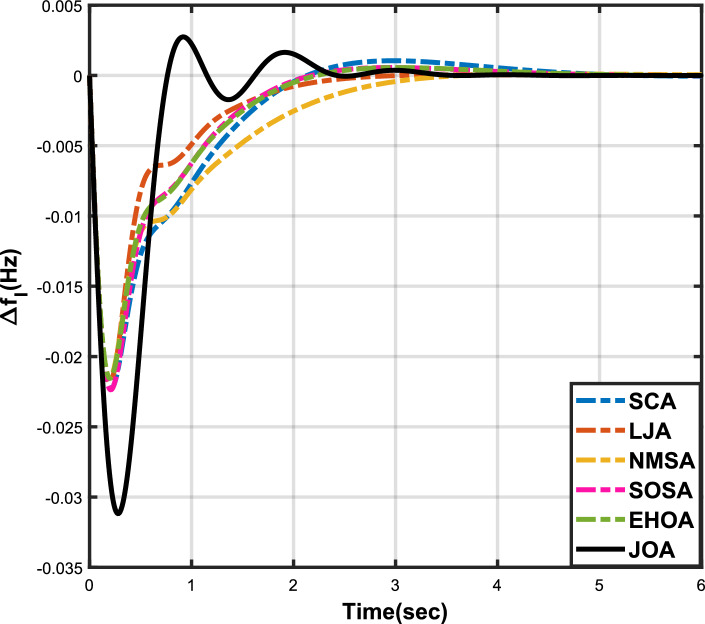
Case 1: Frequency fluctuations for area-1.

**Figure 4:**
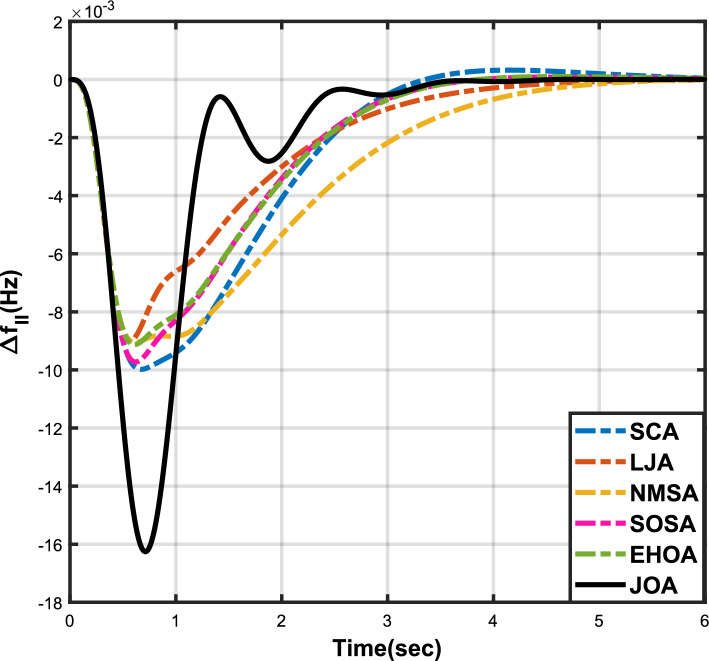
Case 1: Frequency fluctuations for area-2.

**Figure 5:**
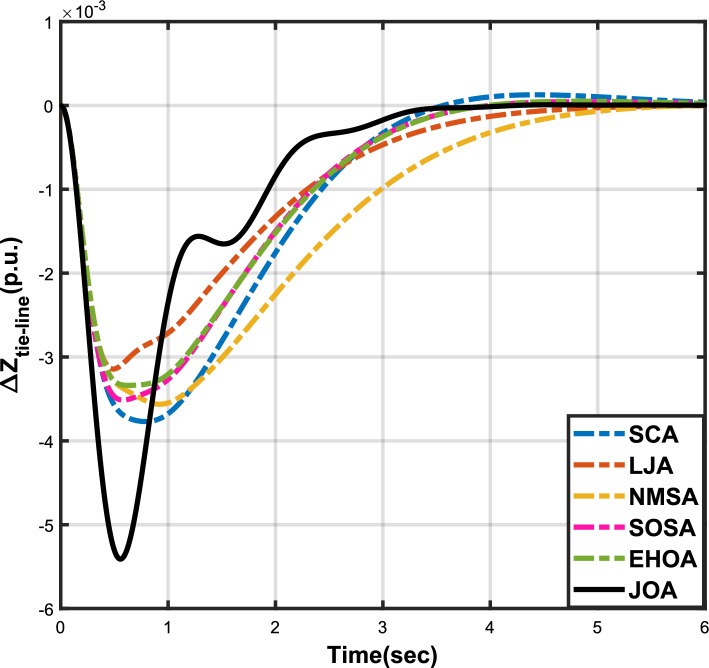
Case 1: Tie-line power fluctuation.

**Figure 6:**
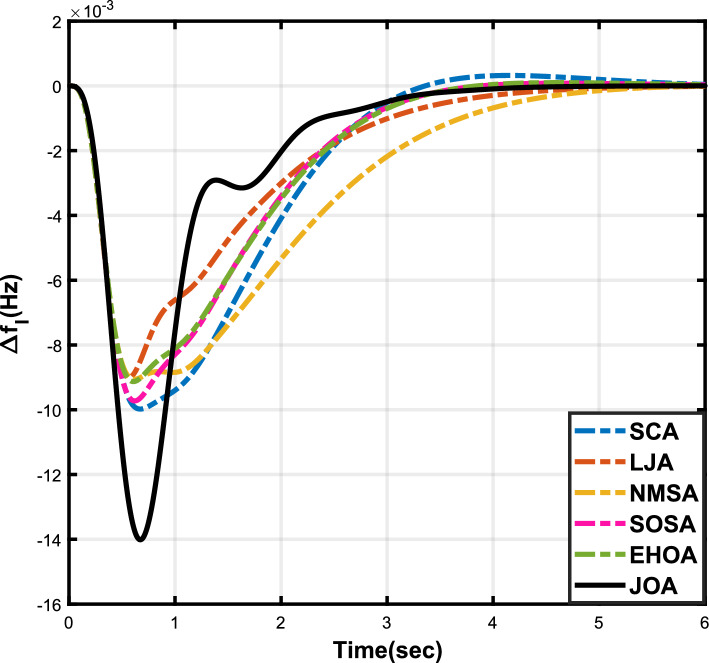
Case 2: Frequency fluctuations for area-1.

**Figure 7:**
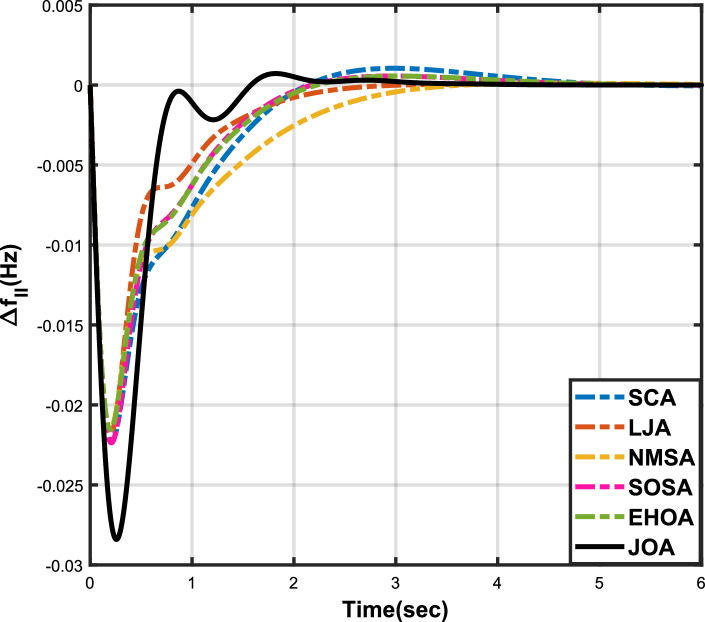
Case 2: Frequency fluctuations for area-2.

**Figure 8:**
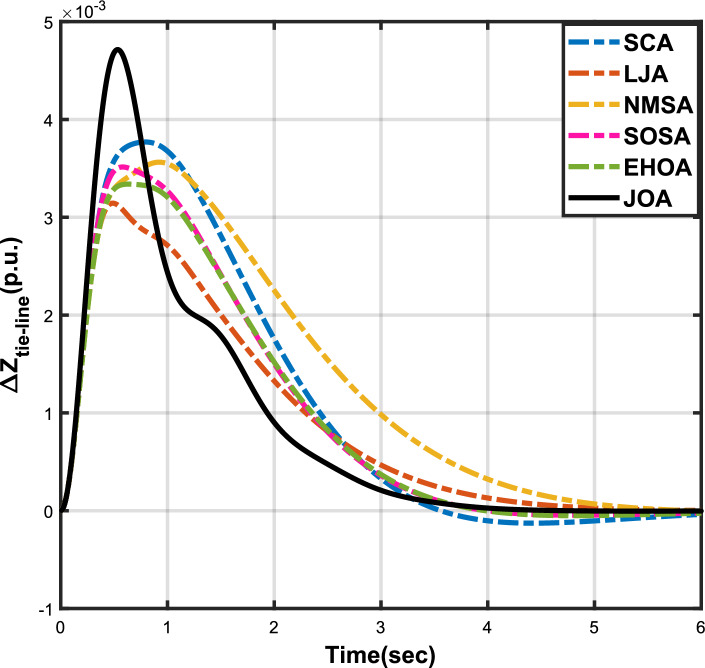
Case 2: Tie-line power fluctuation.

**Figure 9:**
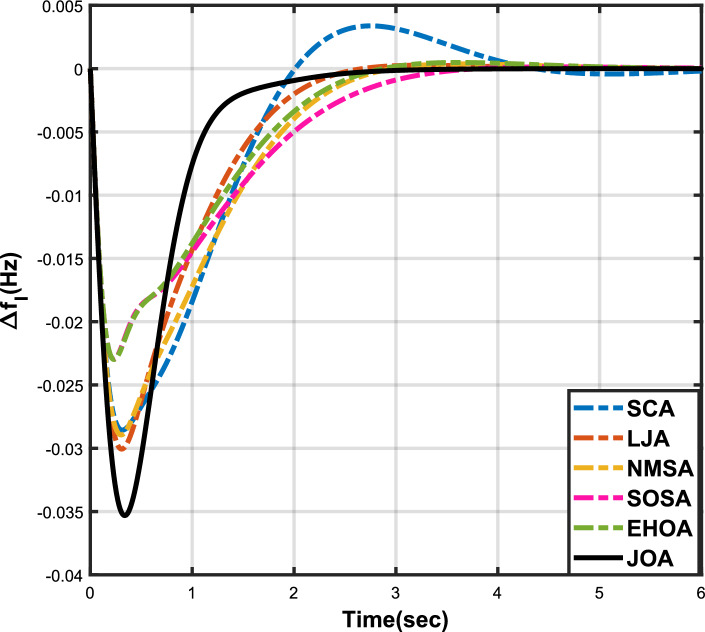
Case 3: Frequency fluctuations for area-1.

**Figure 10:**
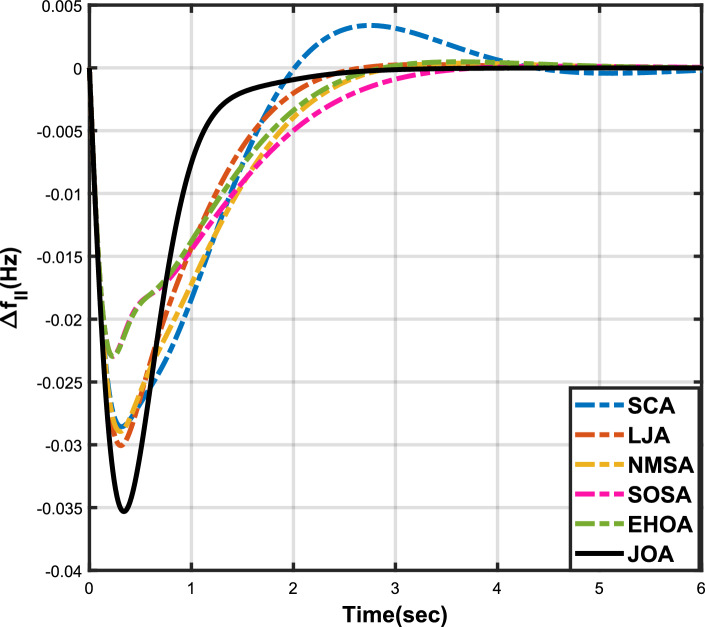
Case 3: Frequency fluctuations for area-2.

**Figure 11:**
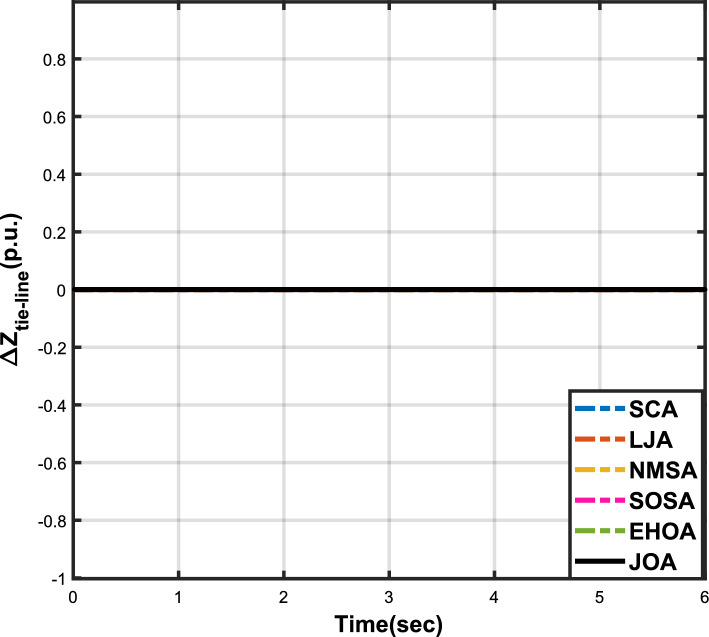
Case 3: Tie-line power fluctuation.

**Figure 12:**
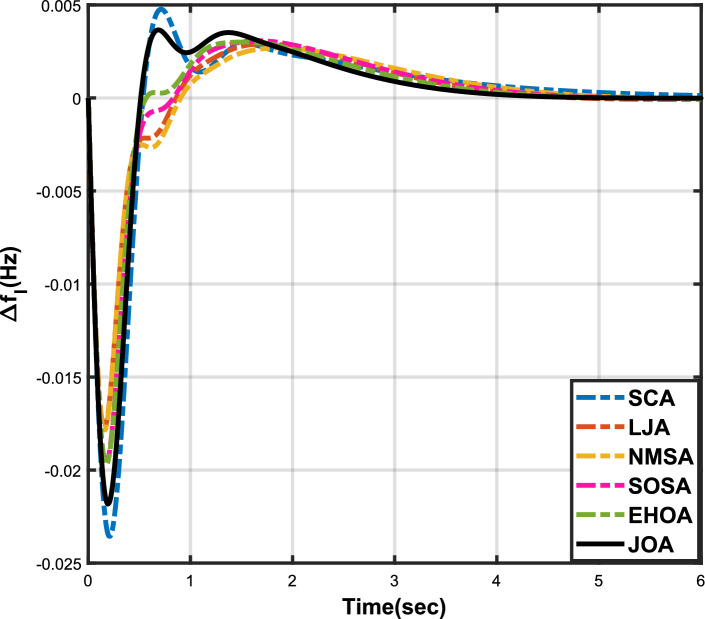
Case 4: Frequency fluctuations for area-1.

**Figure 13:**
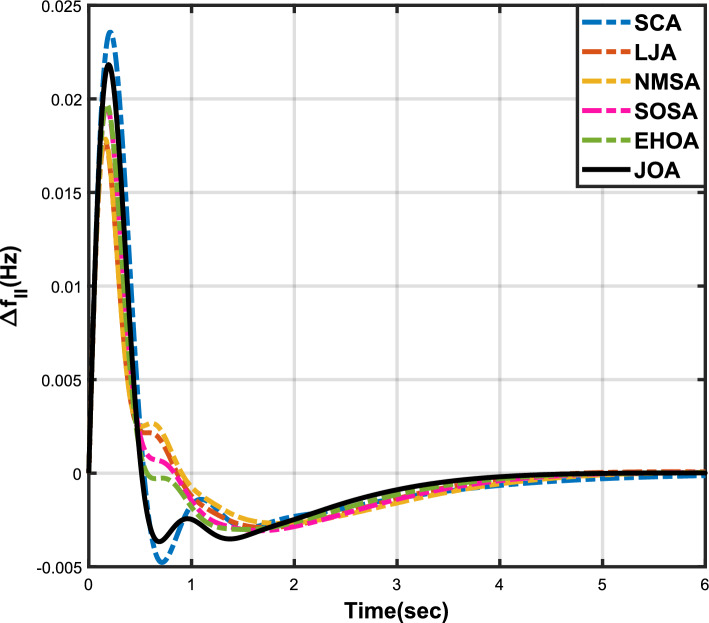
Case 4: Frequency fluctuations for area-2.

**Figure 14:**
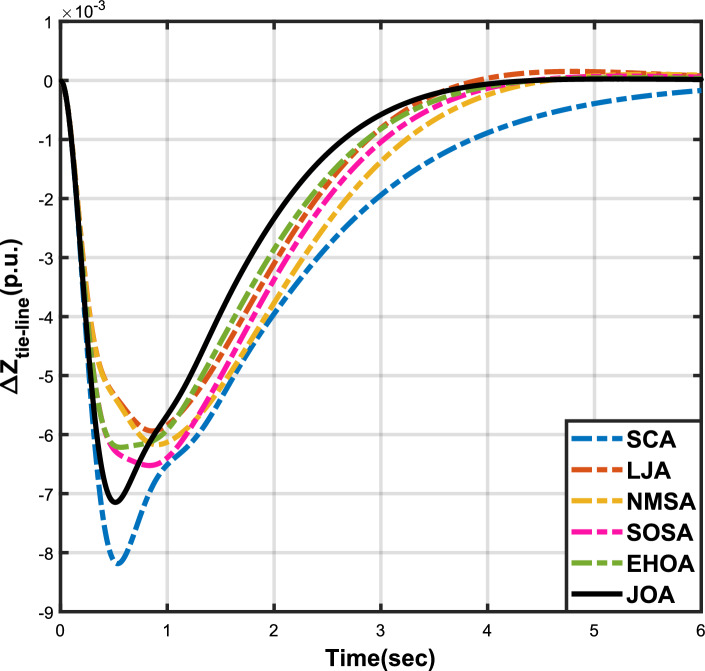
Case 4: Tie-line power fluctuation.

**Figure 15:**
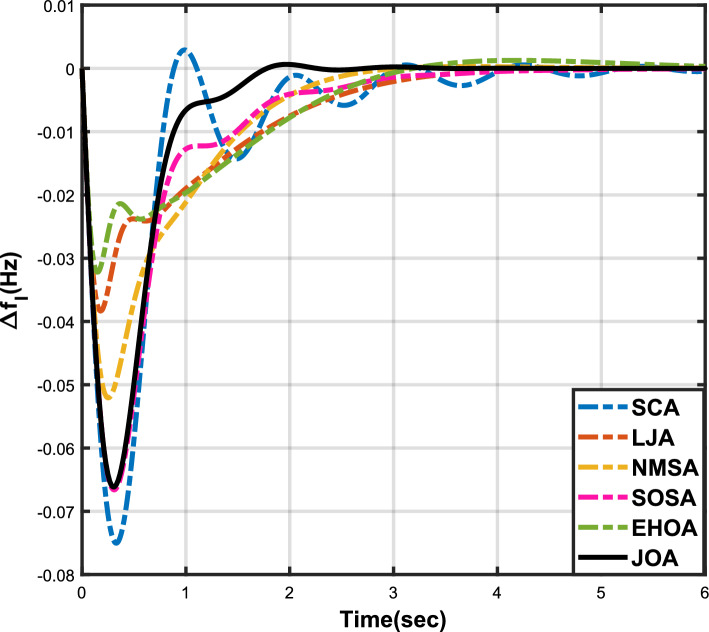
Case 5: Frequency fluctuations for area-1.

**Figure 16:**
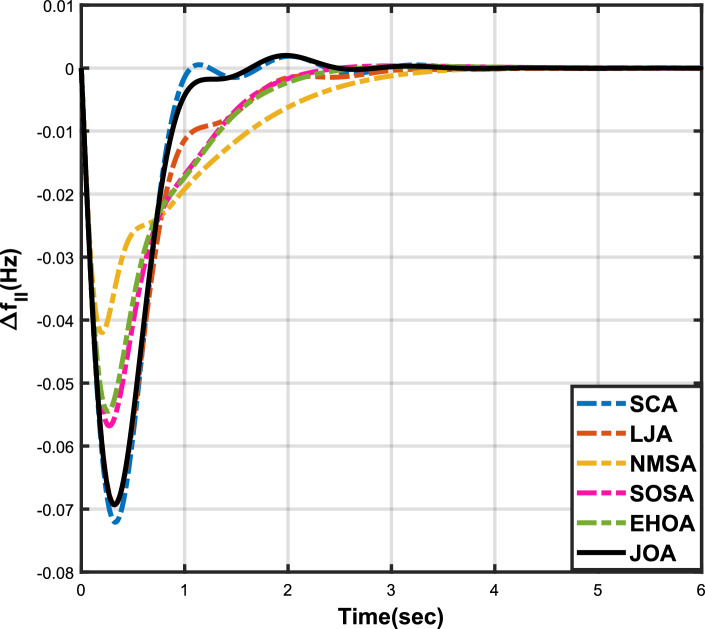
Case 5: Frequency fluctuations for area-2.

**Figure 17:**
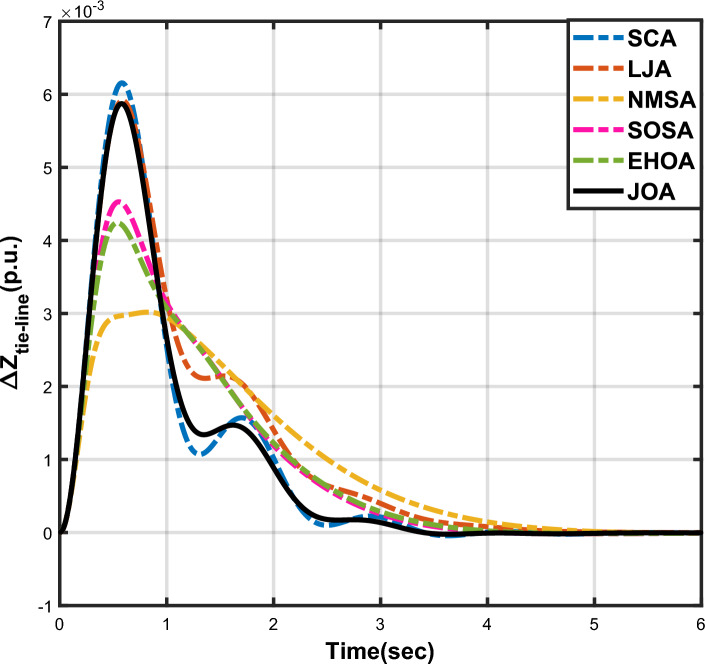
Case 5: Tie-line power fluctuation.

**Figure 18:**
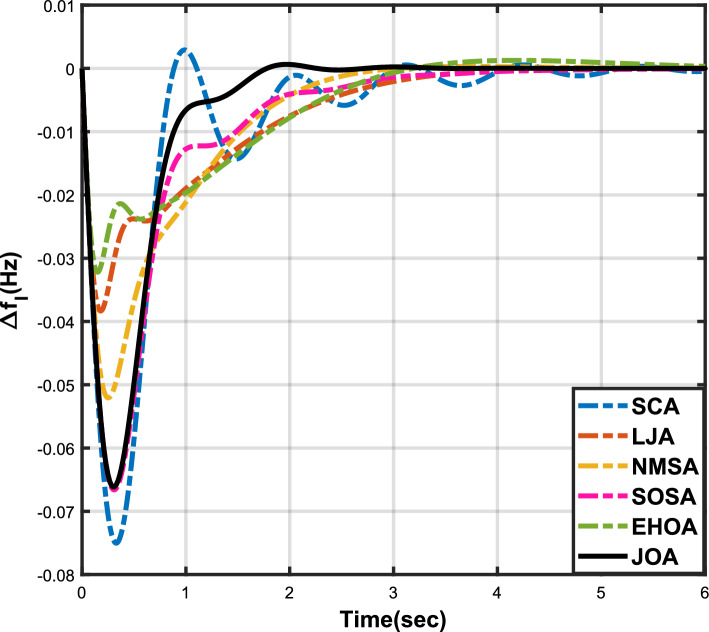
Case 6: Frequency fluctuations for area-1.

**Figure 19:**
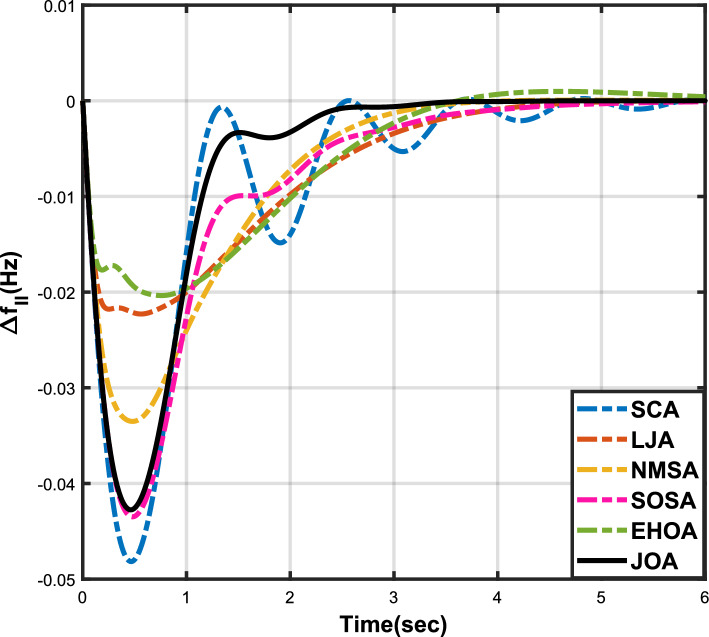
Case 6: Frequency fluctuations for area-2.

**Figure 20:**
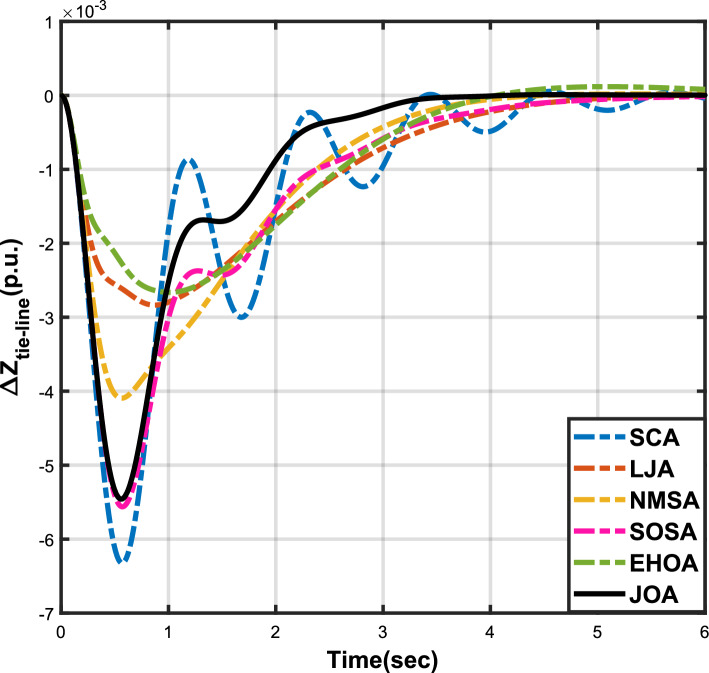
Case 6: Tie-line power fluctuation.

## Results and discussions

This work is expanded on the framework developed by Ali and Abd-Elazim^36^ by analysing a 2-APS. Table 6 outlines the constraints that control the controller parameters. However, (23) provides specifics on the overall objective function that is intended to be minimised. The boundary conditions for these controller parameters are expressed in (24). Table 8 details the step load disturbance assigned to each area in each of the six experimental cases that are investigated to thoroughly evaluate the effectiveness of the FAHP method-assisted PID controller.

The numerical data obtained after performing the simulations for experimental cases I to VI are provided in Tables 9, 10, 11, 12, 13 and 14, respectively, for analytical discussion. Each table provides the most fitted values of objective function (*OF*), sub-objective functions ($OF_{I}$, $OF_{II}$ and $OF_{III}$), and controller parameters ($\Gamma _P$, $\Gamma _I$, $\Gamma _D$ and F), obtained by implementing JOA, SCA, LJA, NMSA, SOSA and EHOA, for the corresponding experimental case. Along with these, the settling time and peak overshooot values of responses (Fig. 3– 20) indicating frequency deviation in area 1 and area 2, and tie-line power deviation, for corresponding experimental cases, are also tabulated in each table.

The outcomes of experimental cases I and II are tabulated in Tables 9 and 10, respectively. The least value (i.e. optimum value) of $OF_{I}$, $OF_{II}$, $OF_{III}$ and *OF* is achieved by JOA, followed by LJA, for both cases. For case I and case II, the responses for frequency deviation in area 1 is shown in Fig. 3 and 6, respectively, and the responses for frequency deviation in area 2 are shown in Fig. 4 and 7, respectively. It can been seen from the responses that, for both cases, the response shown by JOA settles faster than the other algorithms. Similar outcomes is seen for tie-line deviation of case I (Fig. 5) and case II (Fig. 8). The exact settling time values can be seen in Table 9 and Table 10. So, for case I and case II, JOA is proved most efficient among all the considered algorithms.

Tables 11 and 12 provide a summary of the outcomes of experimental cases III and IV, respectively. In both situations, JOA showcased lowest (i.e., optimal) values for $OF_{I}$, $OF_{II}$, $OF_{III}$, and *OF*, followed by LJ algorithm in case III and EHO algorithm in case IV. The frequency deviation responses for area 1 in cases III and IV are shown in Fig(s). 9 and 12, respectively, and those for area 2 are shown in Fig(s). 10 and 13. These responses clearly show that the JOA settles more quickly than other algorithms in both scenarios. The tie-line deviation responses for cases III (Fig. 11) and IV (Fig. 14) show a similar trend. Table 11 and Table 12 tabulated their specific settling time values. From the discussion, it is clear that JOA outperformed all the other algorithms for case III and case IV.

The results of experimental cases V and VI are outlined in Tables 13 and 14, respectively. The optimal values for $OF_{I}$, $OF_{II}$, $OF_{III}$, and *OF* are displayed by the JOA in both cases. In case V, the NMS algorithm came in second, and in case VI, the SOSA algorithm performed better after JOA. Fig(s). 15 and 18, respectively, display the frequency deviation responses for area 1 in cases V and VI, while Fig(s). 16 and 18 display the responses for area 2. These plots conclusively show that in both scenarios, the JOA settles earlier than alternative algorithms. An analogous pattern can be seen in the tie-line deviation responses for cases V (Fig. 17) and VI (Fig. 20). Tables 13 and 14 tabulated the values of their respective settling times. Similar to all the above cases, even for these cases, JOA performed the best among all the algorithms.

A statistical analysis is provided in Table 15. This table tabulates mean ($OF_{Mean}$), minimum ($OF_{Min}$), maximum ($OF_{Max}$) and standard deviation ($OF_{SD}$) values of objective function (*OF*) obtained from JOA, SCA, LJA, NMSA, SOSA and EHOA, for all six cases. From Table 15, it is observed that, JOA is providing the lowest values for $OF_{Mean}$, $OF_{Min}$, $OF_{Max}$ and $OF_{SD}$ for all six cases. The outcome performance of other algorithms vary throughout all the six cases, while JOA is seen being consistent by maintaining the least $OF_{Mean}$, $OF_{Min}$, $OF_{Max}$ and $OF_{SD}$ values for case I to case VI.

**Table 15:** Statistical analysis.

Cases	Statistical measures	JOA	SCA	LJA	NMSA	SOSA	EHOA
I	$OF_{Mean}$	0.01851	0.06187	0.03980	0.05172	0.02877	0.03107
	$OF_{Min}$	0.01770	0.03335	0.02262	0.04087	0.02609	0.02641
	$OF_{Max}$	0.01972	0.08366	0.06050	0.07857	0.03088	0.03842
	$OF_{SD}$	0.00078	0.01903	0.01637	0.01621	0.00191	0.00505
II	$OF_{Mean}$	0.01809	0.06187	0.03980	0.05172	0.02877	0.03107
	$OF_{Min}$	0.01731	0.03335	0.02262	0.04086	0.02609	0.02641
	$OF_{Max}$	0.02005	0.08366	0.06050	0.07857	0.03088	0.03842
	$OF_{SD}$	0.00112	0.01903	0.01637	0.01621	0.00191	0.00505
III	$OF_{Mean}$	0.03010	0.10148	0.06093	0.06738	0.05808	0.05324
	$OF_{Min}$	0.02541	0.06769	0.03822	0.05151	0.05128	0.04371
	$OF_{Max}$	0.03570	0.16136	0.07977	0.09141	0.06340	0.05648
	$OF_{SD}$	0.00384	0.03801	0.01776	0.01554	0.00517	0.00538
IV	$OF_{Mean}$	0.02427	0.05045	0.03373	0.04014	0.03201	0.02837
	$OF_{Min}$	0.02403	0.03794	0.02752	0.03111	0.02888	0.02533
	$OF_{Max}$	0.02455	0.06513	0.04003	0.04977	0.03488	0.02965
	$OF_{SD}$	0.00021	0.01237	0.00517	0.00785	0.00274	0.00175
V	$OF_{Mean}$	0.04136	0.11366	0.09628	0.14511	0.06885	0.08344
	$OF_{Min}$	0.03877	0.04128	0.06272	0.07654	0.05452	0.05560
	$OF_{Max}$	0.04840	0.20169	0.11964	0.21617	0.07725	0.10943
	$OF_{SD}$	0.00401	0.06367	0.02384	0.06441	0.00881	0.01955
VI	$OF_{Mean}$	0.05199	0.12077	0.14000	0.15824	0.11729	0.14098
	$OF_{Min}$	0.04058	0.08797	0.08657	0.07043	0.08278	0.09451
	$OF_{Max}$	0.06674	0.16411	0.18970	0.22823	0.13531	0.18131
	$OF_{SD}$	0.01007	0.02866	0.03758	0.06355	0.02080	0.03328

For further clarification of efficacy and accuracy of the outcomes obtained, Friedman rank test is carried out for JOA, SCA, LJA, NMSA, SOSA and EHOA. This test provides a non-parametric analysis, by allocating a mean rank to all of the six algorithms, and a overall *Q* value and *p* value. The outcomes are said to be verified when *Q* value is positive and *p* value is lesser than 5%. The algorithm attaining the mean rank 1 is considered to be the best among all the algorithms. Table 16 tabulates the results of Friedman rank test. The mean ranks for JOA, SCA, LJA, NMSA, SOSA and EHOA are 1, 4.83333, 3, 5, 3.33333 and 3.83333, respectively. This shows that JOA yeilds best performance, followed by LJA, SOSA, EHOA, SCA and NMSA. The *Q* value came out to be 18.28571, which is a positive value, and *p* value is 0.002609 which is significantly lesser than 5%. Hence these results also provide the clarity on the applicability and efficacy of Jaya algorithm.

**Table 16:** Friedman rank test.

Friedman rank test						
	JOA	SCA	LJA	NMSA	SOSA	**EHOA**
Mean rank	1	4.83333	3	5	3.33333	3.83333
*Q* value	18.28571					
*p* value	0.002609					

## Conclusion

Assessing several attributes simultaneously, figuring out how important each factor is, and choosing a suitable method to compute the weights have always been a matter of concern for decision maker. MADM techniques have helped decision makers to deal with these concerns. One such MADM technique, i.e. FAHP technique is utilized in this contribution to determine weights corresponding to sub-objective functions. The ITAE evaluations of frequency deviations, control errors, and lie-line power deviation for AGC problem of 2-APS are taken into consideration for sub-objective functions. These sub-objective functions are employed for the design of PID controller. The objective function, constructed by combining all weighted sub-objectives, is then minimized using the JOA. The JOA’s efficacy is assessed across six distinct load scenarios. Optimization is also carried out using SCA, LJA, NMSA, SOSA, and EHOA to show the efficacy of the JOA-based controller. For each of the six load variations, their results are compared, and the comparisons are shown in tabular and graphical form. Specifications like peak overshoots, settling time, decision parameters, and objective function values are compared. The outcomes showed that for all load variations taken into consideration, the JOA consistently performs better than the other algorithms. Furthermore, Friedman rank test and statistical analysis support the superiority of the JOA-based PID controller over alternative controllers.

## Data Availability

The datasets used and/or analyzed during the current study are available from the corresponding author upon reasonable request.
